# Utilizing a Human–Computer Interaction Approach to Evaluate the Design of Current Pharmacogenomics Clinical Decision Support

**DOI:** 10.3390/jpm11111227

**Published:** 2021-11-18

**Authors:** Amanda L. Elchynski, Nina Desai, Danielle D’Silva, Bradley Hall, Yael Marks, Kristin Wiisanen, Emily J. Cicali, Larisa H. Cavallari, Khoa A. Nguyen

**Affiliations:** 1 Department of Pharmacotherapy and Translational Research, College of Pharmacy, University of Florida, Gainesville, FL 32611, USA; elchynskia@archildrens.org (A.L.E.); desainina@ufl.edu (N.D.); ddsilva@ufl.edu (D.D.); bhall106@ufl.edu (B.H.); yael.marks@ufl.edu (Y.M.); kwiisanen@cop.ufl.edu (K.W.); emily.cicali@cop.ufl.edu (E.J.C.); lcavallari@cop.ufl.edu (L.H.C.); 2 Center for Pharmacogenomics and Precision Medicine, University of Florida, Gainesville, FL 32611, USA; 3 Arkansas Children’s Hospital, Little Rock, AR 72202, USA

**Keywords:** clinical decision support, pharmacogenomics, human–computer interaction, usability evaluation, formal assessment

## Abstract

A formal assessment of pharmacogenomics clinical decision support (PGx-CDS) by providers is lacking in the literature. The objective of this study was to evaluate the usability of PGx-CDS tools that have been implemented in a healthcare setting. We enrolled ten prescribing healthcare providers and had them complete a 60-min usability session, which included interacting with two PGx-CDS scenarios using the “Think Aloud” technique, as well as completing the Computer System Usability Questionnaire (CSUQ). Providers reported positive comments, negative comments, and suggestions for the two PGx-CDS during the usability testing. Most provider comments were in favor of the current PGx-CDS design, with the exception of how the genotype and phenotype information is displayed. The mean CSUQ score for the PGx-CDS overall satisfaction was 6.3 ± 0.95, with seven strongly agreeing and one strongly disagreeing for overall satisfaction. The implemented PGx-CDS at our institution was well received by prescribing healthcare providers. The feedback collected from the session will guide future PGx-CDS designs for our healthcare system and provide a framework for other institutions implementing PGx-CDS.

## 1. Introduction

Pharmacogenomics clinical decision support (PGx-CDS) is an essential tool to advance the adoption of clinical pharmacogenomics in health systems across the country [1,2]. Clinical decision support (CDS) tools enhance medical decision-making by providing relevant information about the patient (e.g., renal function, drug–drug interactions) at the time of prescribing to improve providers’ clinical workflow and, in turn, potentially improve patient outcomes [3,4,5]. Prescribers encountering pharmacogenomic (PGx) results often require clinical resources to interpret results and apply them to prescribing decisions in large part because of limited formal PGx education in professional schools [6,7,8]. PGx-CDS helps guide healthcare providers, including, but not limited to, physicians, pharmacists, and nurse practitioners, in making decisions regarding pharmacotherapy based on PGx information for their patients [9,10].

One of the major gaps with the current PGx-CDS is the lack of formative assessment of provider needs [11,12]. If PGx-CDS does not aggregate necessary information from electronic health records (EHRs) and fit within the natural workflow of prescribers, it can introduce errors, promote alert fatigue, and potentially cause frustration for the end-user [13]. Human–computer interaction (HCI) is a multidisciplinary field of formative assessment that focuses on the interaction between humans, or users, and computers. It aims to improve the design, implementation, and evaluation of computer systems for human use. HCI has been utilized successfully in the development of many CDS tools at many different stages [14,15]. For instance, Khan et al. utilized interview and survey results to develop a CDS tool to be used by emergency healthcare providers to help stratify a patient’s risk for a pulmonary embolism [16]. Interestingly, their results showed that such CDS tool was more relevant as a point-of-care tool generated from an order entry instead of a nurse triage trigger in the emergency department. This signifies the importance of using formative assessment to identify the right workflow for CDS tools before development. Fossum et al., at the other spectrum of formal assessment, utilized cognitive walkthrough and usability evaluation to develop CDS for treating pressure ulcers and malnutrition in nursing care residents [17]. Utilizing group interviews, cognitive walkthrough observations, and usability evaluation, they were able to identify key facilitators of CDS use, including ease of use, and a supportive work environment. In the PGx-CDS area, Nguyen et al. developed a prototype to assess thiopurine PGx-CDS utilizing interviews and usability evaluation [10]. The prototype received high satisfaction scores from providers since it was developed and tested based on providers’ feedback. Finally, Dolin et al. successfully developed a functional prototype PGx-CDS that can look through genetic data in a Genomic Archiving and Communication System [18] using Fast Healthcare Interoperability Resources (FHIR) and the CDS Hooks technique. Overall, HCI was positively and effectively utilized at the early phase of PGx-CDS development. However, there is a lack of formal assessment on PGx-CDS tools developed and implemented in EHR systems. Therefore, the objective of this study was to evaluate the usability of PGx-CDS tools that have been implemented in a healthcare setting.

## 2. Materials and Methods

### 2.1. Study Setting

We conducted a usability evaluation at the University of Florida Health Shands Hospital, a large academic medical center with EPIC^®^ as the EHR. PGx-CDS was implemented at the hospital initially in 2012 and was led by the UF Health Precision Medicine Program, a team of interdisciplinary experts. All study procedures were conducted in a test environment of the EHR. The study was approved by the University of Florida Institutional Review Board.

### 2.2. Participant Recruitment

We intended to recruit ten participants for this study. We utilized a rolling recruitment process in which we sent emails to ten random providers who were eligible to participate from the health system. After a follow-up email one week later, we sent emails to another batch of ten providers. This process was repeated until we were able to conduct 60-min usability tests on ten participants. A total of 74 emails were sent to our potential participant pool. Each participant verbally consented prior to the session through a secure online video chat. Signed consent forms were then collected through email.

### 2.3. Scenario Development

**Figure 1:**
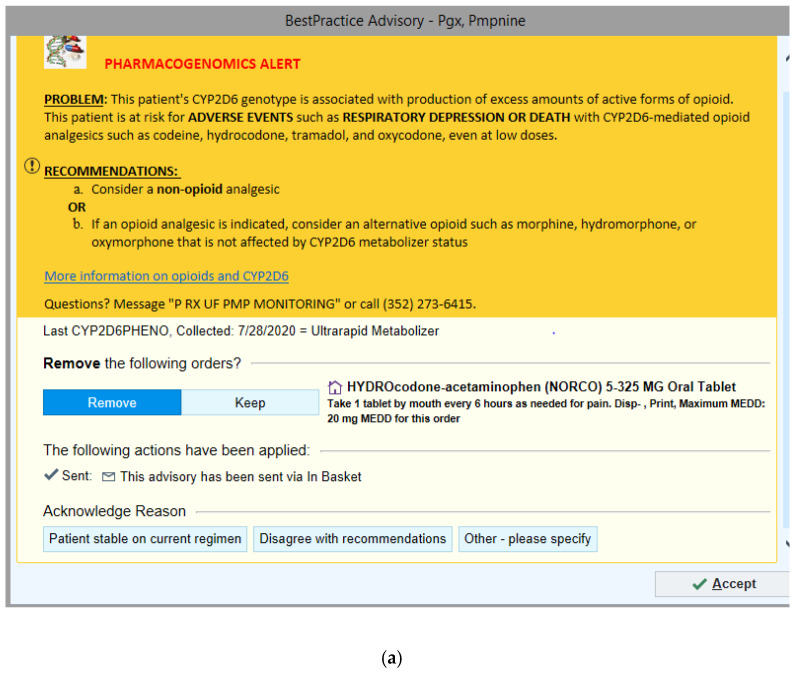

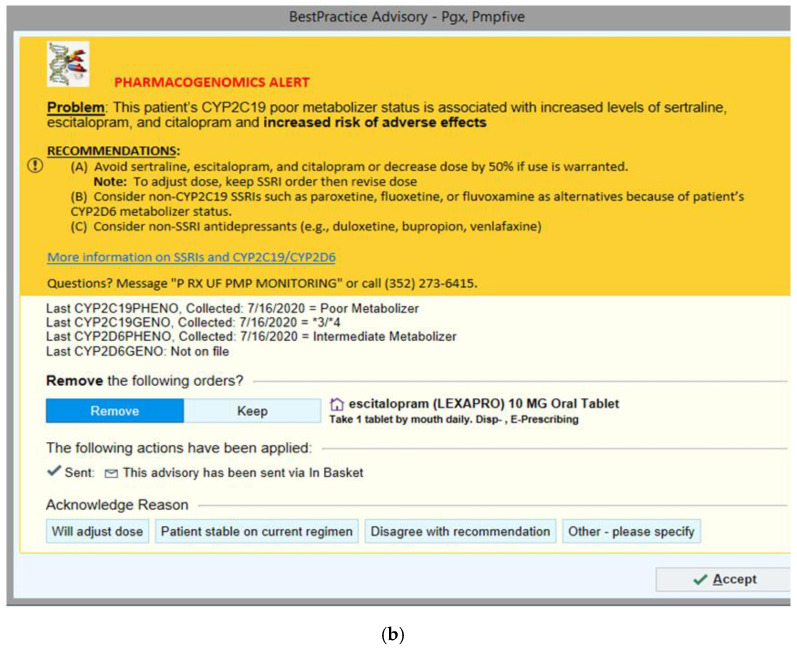
Pharmacogenomics-Clinical Decision Support (PGx-CDS) that fired during the two clinical scenarios. (**a**) Hydrocodone-acetaminophen prescribed to a patient with a CYP2D6 ultrarapid metabolizer (UM) phenotype; (**b**) escitalopram prescribed to a patient with CYP2D6 intermediate metabolizer (IM) and CYP2C19 poor metabolizer (PM) phenotypes.

Standardized patient scenarios were developed by a team consisting of an informatics pharmacist, a pharmacogenomics specialist, a pharmacogenomics pharmacy fellow, and pharmacy students. Scenarios were adapted from clinical scenarios to closely simulate the clinical setting and utilize already implemented PGx-CDS at UF Health (Figure 1a,b). The two scenarios developed were (1) hydrocodone-acetaminophen prescribed to a patient with a CYP2D6 ultrarapid metabolizer (UM) phenotype and (2) escitalopram prescribed to a patient with CYP2D6 intermediate metabolizer (IM) and CYP2C19 poor metabolizer (PM) phenotypes. When a provider orders a relevant medication (hydrocodone-acetaminophen for scenario 1 and escitalopram for scenario 2) in the EPIC^®^ EHR, it triggers an active PGx-CDS alert. A more detailed description of the two scenarios can be found in Supplemental Material File S1.

### 2.4. Simulation Procedure

**Figure 2:**
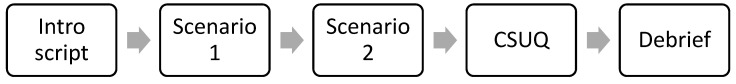
Chronological order of the simulation procedure completed by the healthcare providers during the 60-min usability session. CSUQ = Computer System Usability Questionnaire.

Figure 2 provides the chronological order of the 60-min simulation procedure and was applied to all participants. Three moderators conducted all usability sessions for the ten providers. For each session, the moderator read a scripted introduction to maintain consistency across sessions. Participants were informed of the goal of the usability session, their tasks, and the procedures. They were asked to complete patient scenarios using a test EHR environment. Participants were asked to “think aloud” by verbalizing their thoughts and reactions as they interacted with the alerts while completing the two scenarios. Subsequently, participants completed the Computer System Usability Questionnaire (CSUQ), a validated computer usability satisfaction questionnaire, via REDCap. Finally, the moderator conducted a debrief interview to collect additional feedback and comments about the interface and interaction (Supplemental Material File S2).

No time limit was imposed for completing tasks related to each scenario and filling out the CSUQ. Because of restrictions related to the COVID-19 pandemic, all sessions were recorded and conducted online using secured video conference software, Zoom (San Jose, CA, USA). The moderators opened the EHR on their monitors, shared their screen, and gave the participants control to allow the participants to perform their tasks.

### 2.5. Data Collection and Outcome Measures

Qualitative usability data were collected via the “Think Aloud” technique, with participants’ verbalizations captured by screen video and audio recordings during the session. Additionally, qualitative data, including information from debriefing interviews, were captured and collected. Two analysts reviewed the video and audio recordings individually to capture the main content of each usability session. A priori code was adopted from Richardson et al., usability, visibility, workflow, content, understandability, medical usefulness, and navigation, except for practical usefulness [19] (Table 1). The team then sorted participants’ comments into themes relevant to each code that could be classified into three categories: positive, negative, and suggestion. The research team discussed each theme to reach consensus on categorization. Finally, the CSUQ 19-item questionnaire evaluated participant satisfaction with the PGx-CDS. In addition to capturing participant overall perceptions of satisfaction of PGx-CDS, this questionnaire assessed their satisfaction in three main domains: system usefulness (how meaningful the PGx-CDS is to providers), information quality (the value of information presented in the CDS), and interface quality (the condition of information displayed in the PGx-CDS). Quantitative data included demographics and time to complete each scenario, including ordering the medication in the EHR and interacting with the relevant CDS.

## 3. Results

### 3.1. Participant Characteristics

All ten participants were physicians, with a mean age of 37 ± 10 years; 60% were residents, 60% were female, and 70% were white. Seventy percent of the participants reported seeing PGx-CDS less than once a month. All participants had over one year of experience working with EPIC^®^, and none reported visual impairment or color blindness (Table 2).

### 3.2. Satisfaction

**Figure 3:**
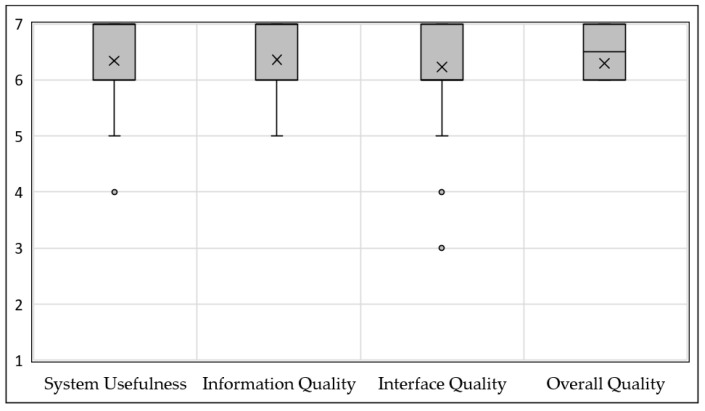
CSUQ box-whisker plot.

Satisfaction with the PGx-CDS was high across participants. The mean CSUQ score for overall satisfaction was 6.3 ± 0.95, with seven strongly agreeing and one strongly disagreeing for the PGx-CDS. System usefulness, information quality, and interface quality had mean scores of 6.3 ± 0.76, 6.4 ± 0.90, and 6.2 ± 0.97, respectively (Figure 3).

Seven is strongly agreeing with statements and one is strongly disagreeing with the statements. The “x” is the mean of the ten participants.

### 3.3. Efficiency

**Figure 4:**
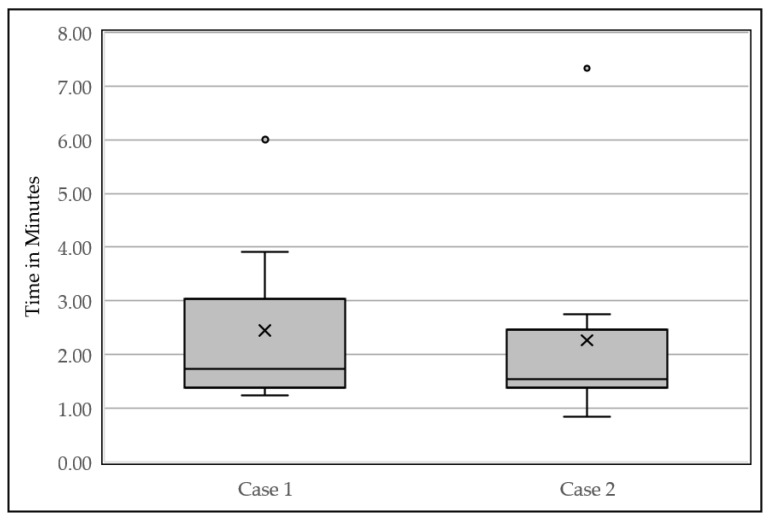
Time to complete each of the clinical scenarios box-whisker plot. The median time to complete scenario one was 1.73 min, and scenario two was 1.54 min. The “x” is the mean of the ten participants.

All ten participants completed both assigned scenarios from the usability test with minimal help from the moderator. Specifically, participants mainly requested assistance with finding the test patients, or opening a new encounter. The median time to complete scenario one was 1.73 min, and scenario two was 1.54 min (Figure 4).

### 3.4. Emerging Themes Identified from the Usability Session

Table 3 summarizes emerging themes analyzed during the usability sessions with relevant example quotes collected. The themes were then categorized into seven codes. Their definitions are included in Table 1.

#### 3.4.1. Usability

As indicated in the satisfaction score, the alerts were favorably viewed since they were similar to other non-PGx CDS already employed in the health system (“that’s standard for how I currently use EPIC^®^” participant 07). In addition, the recommendation was clear for participants with less knowledge of PGx. Participant 03 commented, “It seems pretty user-friendly even for someone who is not experienced in pharmacogenomics.” However, many users felt the “more information” button was not intuitive. The button was a hyperlink in the CDS that allowed participants to review references for PGx recommendations.

For suggestions to improve usability, several participants recommended linking alternative medications in the CDS to minimize the number of steps the providers need to complete to prescribe an appropriate medication. Participant 01 recommended, “It’s a little bit cumbersome to leave that [alert] screen… [it’s] easier if there were links to orders.” Similarly, a participant suggested an option to link to the lab section within the patient’s EHR because “I don’t necessarily know how to find them [PGx lab results]… [it] can be hard to find labs in EPIC^®^ and so link to them would be nice” (Participant 03 comment).

#### 3.4.2. Visibility

Many providers agreed that having yellow sections alerted the user to important information (“the yellow box [on] top -fantastic. Very to the point”, Participant 01) and having an image of DNA helped alert the provider that it is a PGX-CDS from other types of CDS (“I like that it says the ‘Pharmacogenomics Alert’ and then the picture of the DNA”, Participant 02). However, when reading the PGx-CDs interface, providers found the lab reporting system in the CDS was poorly presented and that it was hard to recognize the patient’s phenotype. Participant 06 stated, “I read things kind [of] quickly and the ‘poor metabolizer’ [a type of phenotype] doesn’t jump out at me.” Suggestions to improve the CDS visibility included bolding the patient’s phenotype results, moving the lab results into the yellow sections, or adding more spaces between each row.

#### 3.4.3. Workflow

The workflow of PGx-CDS played an important role in provider satisfaction. Providers appreciated that the alerts were designed as an active alert (hard stop) when the provider prescribes the relevant drug (“I like the fact that it clearly grabs your attention as soon as you try to order the medication… and there is an actual stop in the workflow”, Participant 03.) The “hard stop” is necessary for providers to read relevant content to make an optimal prescribing decision. However, one participant recommended having the CDS alerts only when a new medication is prescribed (“I’d be worried about getting fatigued… so I think anytime I add a new drug I think it is appropriate if it applies but dosing changes, I don’t think I would want them to pop up every time”; Participant 06 in response to dose decrease recommendation in the escitalopram alert). Additionally, providers recommended including an option to document their interactions/changes from the PGx-CDS in the EHR system. For instance, Participant 10 suggested, “I want [this] information to retain in EPIC^®^. It gives me an opportunity to document my change”.

#### 3.4.4. Content

The systematic CDS format (Figure 1) received high praise from participants (“The information is fantastic… I think really has the potential to cut down on adverse effects”, Participant 01.) Alternative suggestions were also useful to help providers make changes. Providers who were not familiar with PGx content did not see the need to provide genotype information in the CDS. Participant 07 commented, “I don’t think the genotype is useful for me whatsoever, I’m not [going to] know what the *3/*4 genotype actually means.” However, other providers wanted to see both genotype and phenotype information (“I want to know the actual genetic phenotype and genotype”, Participant 10). Content’s related recommendations from providers included adding a “button” to request a consultation and providing an option to order an alternative.

#### 3.4.5. Understandability

Understandability of the problem statements and recommendations was clear and easy to understand to all providers. Participant 02 commented, “What I get from that is… she does not metabolize it as fast or basically different from other people, so she basically more sensitive” when s/he was asked about the problem statement. Still, many participants were not able to recognize and interpret PGx laboratory results easily. Participant 01 was concerned, “I didn’t realize necessarily that they were labs. I’m not familiar with these phenotypes and genotypes.” Additionally, the problem statement was not clear when two genes were used to provide a recommendation for antidepressant therapy. Therefore, providers recommended making the metabolizer status associated with the gene–drug pair clearer.

#### 3.4.6. Medical Usefulness

Providers agreed the alerts are medically useful and helped them review the appropriateness of their medication selection (“This message has me thinking as to what I should do”, Participant 02) and guided the change in their order (“It [PGx-CDS] definitely made me think. It made me go back and change my order”, Participant 01). A participant also requested that a list of potential side effects be provided for each gene–drug pair to determine the clinical importance. The participant stated, “I would want to know what to look out for as a reminder from medical school because that’s the last time I would have gone through the side effects” (Participant 06). Additionally, they wanted to include both generic and brand names because “people might not be as familiar with the non-brand name,” Participant 05 suggested.

#### 3.4.7. Navigation

Recommendations by participants to improve the navigation in the CDS were conflicting. Many providers agreed that navigating the CDS was difficult for the acknowledgment reasons sections and unsure why comments were needed (for acknowledging reasons). For example, participant 03 stated, “I am not really sure what comment they want me to enter.” However, they did not recognize that comments were optional. At the same time, other participants, who were experienced with the EPIC^®^ system, found navigating the alert to be similar to other CDS in the healthcare system and therefore were able to navigate through easily. Participants also suggested hiding the acknowledgment section unless the user selects to continue with the medication that generated the PGx-CDS.

## 4. Discussion

This is one of the first studies that has utilized HCI to evaluate implemented PGx-CDS tools. Overall, we found that PGx-CDS were well-received based on the CSUQ averages and positive feedback from the providers. The average CSUQ score for all categories was more than six (6.3 ± 0.2), with a max score of seven indicating strongly agree. There were several reasons for this high acceptance score. First, our PGx-CDS was designed using the same Best Practice Advisory framework already implemented in the healthcare system, and therefore, providers were familiar with the structure of the alert. As described by Jakob’s law, “consistency and standards” is one of the ten principles of interaction design. The users did not have to wonder or learn the process when interacting with our PGx-CDS alerts because we utilized the same framework and format as other CDS alerts available in the EHR system [20]. Additionally, we implemented a consistent format for all PGx-CDS (problem statement, recommendations, actions, acknowledgment, see Figure 1), thus cutting down on the potential learning curve, as evident by the decrease in the median time it took to complete scenario two (1.54 min) versus scenario one (1.73 min). Second, we deployed standard list acknowledgment options, allowing providers to provide trackable feedback. Overall, from the usability sessions, providers had a common understanding of different acknowledging buttons and when to use each button when encountering the PGx-CDS.

Our study utilized the usability evaluation to collect immediate feedback from the healthcare providers on select implemented PGx-CDS. This method has been used commonly to collect feedback from users [19,21]. However, it has not been applied often to implemented PGx-CDS tools. One similar study is by Devine et al., in which ten physicians who specialized in either cardiology or oncology were enrolled. The physicians were presented with a scenario and an alert that correlated to the case, similar to our study. Providers echoed similar concerns such as the need to include both brand and generic names of the medications, remove genotype information, and the need to simplify explanation resource. Similar positive outcomes to our study were providers liking the explanation of how the drug is metabolized and wanting these alerts to fire on the gene–drug pairs as they found them clinically helpful. Differences between our studies include that the EHR used was Cerner^®^, and our study received higher agreement on the usability survey [21].

Richardson et al. identified similar themes, which included that the CDS was determined to be relevant and accurate for decision making and the added coloring to the CDS improved visibility. Similar suggestions to improve CDS between our studies were refining the intuitiveness for the next steps once an alert was generated and discerning between the options available on the CDS. Richardson’s study, however, assessed a complex scoring tool to calculate the risk for health conditions [19] instead of a CDS. Significant feedback collected in our usability evaluation was related to the display and interpretation of laboratory results. In our current setting, laboratory results were displayed after the problem statement and recommendations, causing confusion and misinterpretation from participants. One change that can be amended is only including the phenotype information in the CDS alert, as many providers did not find the genotype information within the CDS helpful. Participants also suggested including the phenotype result earlier in the PGx alert (for example, within the problem statement) in a more readable format. Finally, the current technology restriction at our local institution only allows us to display phenotype interpretations within the PGx-CDSs or laboratory reports (the latter were buried deep in the patient information with other laboratory results). Therefore, providers might have problems finding these PGx results within the EHR. In fact, many participants raised their concerns about this restriction. The institution is in the process of implementing the EPIC^®^ Genomics module, which will allow us to resolve this problem by displaying all genomics information and interpretation in a specific patient’s genomic profile. Overall, our study focused on possible solutions to practically improve the design and display of PGx results in the EPIC^®^ system.

In addition to a lab results display, participants provided many other suggestions. While many suggestions were beneficial for the enhancement of PGx-CDS, some were not practical for deployment due to the current technology restriction from the EPIC^®^ EHR. This restriction can create a sizable gap between providers’ expectations and technology feasibility. In our study, many participants recommended having an actionable button to help order alternative medication(s). However, this suggestion can be troublesome when there are multiple options available. For instance, our opioid PGx-CDS recommended that providers order non-opioid analgesic medications as an alternative. However, creating an actionable button for non-opioid analgesics is impractical because there are multiple options (e.g., acetaminophen, several NSAIDs, aspirin). In addition, our PGx-CDS only provides PGx specific recommendations. Alternative medication decisions will also depend on other patient factors (e.g., age, allergies, renal function). While current technology in healthcare might not be capable of resolving provider expectations, these suggestions may be applied in the future with advanced technology utilizing artificial intelligence or multi-dimensional CDS to suggest more relevant alternatives.

This study has several limitations. The first limitation, similar to Richardson et al., was that the sample of providers was a convenience sample who potentially had a greater interest in CDS than a random sample [19]. The study was limited by its small sample size, but it has been determined that you only need five participants to identify 85% of the usability errors [22]. The ten participants were all physicians, and thus results may not be generalizable to other healthcare providers, such as advanced practicing registered nurses. We only included two cases for the usability tests. The chosen cases may not apply to all providers, such as an anesthesiologist prescribing new start escitalopram in scenario two, but feedback collected aligned with other physician feedback and suggestions. Lastly, this study was conducted using EPIC^®^ EHR at an academic hospital. Results found may not be generalizable to a nonacademic hospital or an institution using other EHRs.

### Future Directions

The result of this study may be used to enhance the design of currently implemented PGx-CDS at our institution and provide a framework for other institutions who are implementing PGx-CDS. Once the alerts have been updated, we will continuously conduct further usability sessions to evaluate our PGx-CDSs’ functions and satisfaction. Ultimately, practical design guidelines can be developed to assist other institutions with PGx-CDS development.

## 5. Conclusions

Utilizing the “Think Aloud” usability testing and having providers complete the CSUQ following the two PGx-CDS scenarios resulted in invaluable feedback. The feedback collected will guide the changes to the implemented PGx-CDS at our institution and help create a standardized PGx-CDS format.

## Figures and Tables

**Table 1:** Codebook used during the “Think Aloud” session to help categorize the statements the healthcare providers provided on the two PGx-CDS.

Coding Category	Definition
**Usability**	Ease of use of the CDS tool, ability to use with minimal effort. This can be both positive and negative (usability errors).
**Visibility**	Ability to quickly recognize key messages and instructions provided by the CDS tool. Colors and symbols are examples of how messages can be easily recognized in the CDS.
**Workflow**	Ability of the CDS tool to fit into the natural order of events in a patient encounter.
**Content**	Medical accuracy or appropriateness of CDS tool text included as orders, patient information, documentation.
**Understandability**	Ability to quickly comprehend meaning of text, instructions, and the purpose of CDS components.
**Medical Usefulness**	Improves clinical decision-making during encounter.
**Navigation**	Ability to move easily through the CDS tool.

**Table 2:** Characteristics of the ten healthcare providers.

Characteristics	*n* = 10
Female Sex (%)	6 (60)
Race (%)	
*Asian*	2 (20)
*White*	7 (70)
*I prefer not to answer*	1 (10)
Age (avg ± SD)	37 ± 10
Degrees/Certification (%)	
*MD*	9 (90)
*DO*	1 (10)
*Other*	0 (0)
Years working at UF (%)	
*Less than 1 year*	1 (10)
*1–10 years*	8 (80)
*11–20 years*	1 (10)
Years using EPIC^®^ EHR (%)	
*Less than 1 year*	0 (0)
*1–10 years*	9 (90)
*11–20 years*	1 (10)
How often do you encounter PGx BPAs a month (avg)? (%)	
*Less than once a month*	7 (70)
*1–10 times a month*	2 (20)
*11–20 times a month*	1 (10)
Uncorrectable visual impairments or color blindness (%)	
*No*	10 (100)

**Table 3:** Themes collected from the “Think Aloud” session categorized into codes.

Code		Themes	Example
1. Usability	Positive	PGx-CDS developed were user-friendly even for users with less knowledge of PGx. CDS provides clear instruction, so a participant was able to formulate decisions without assistance.	“This one [PGx-CDS] is a lot more user friendly.”—MD03. “It seems pretty user-friendly even for someone who is not experienced in pharmacogenomics.”—MD03. “I think it’s really straight-forward and one of the more usable alerts we get.”—MD04.
		The consistency of CDS’s format across alerts allows participants to learn and adapt fast (learnability).	Participants improved time spent on BPA1 compared to BPA2.
	Negative	Acknowledge reasons mechanism was confusing. The system automatically switched to ‘Keep’ current order (from the default being ‘Remove’) if an acknowledged reason was selected. This issue is not PGx-CDS specific.	The participant was confused by the acknowledge reason section. They did not realize you only fill it out when you elect to keep the ordered medication. The participant filled out ‘Other’ for the acknowledge reason section and did not realize that it changed the order to ‘Keep.’ They were confused after selecting ‘Accept’ when the next page was the medication order screen.
		The “More information” button was not intuitive. Participants were looking for a summary of information from reference pages.	“Using my surgery brain, I’m just looking for the high yield colorful pictures.” Participant was confused on how to navigate within the more info link and eventually gave up, seeming not to find it very useful—MD02.
	Suggestions	Alternative medications recommended should be orderable and should link directly from the CDS to avoid the cumbersome work of creating a new order.	“It’s a little bit cumbersome to leave that [alert] screen… [it’s] easier if there were links to orders.”—MD01.
		There should be a link available to the lab results since they can be hard to find in the HER.	“I don’t necessarily know how to find them [PGx lab results]… can be hard to find labs in EPIC^®^ and so link to them would be nice.” Participant would like a link to help explain the labs more—MD03.
2. Visibility	Positive	Many participants mentioned that the pronounced yellow section (“Problem” and “Recommendations”) help catch their attention.	“The yellow box up top-fantastic. Very to the point.” Referring to content and color of BPA one—MD01.
		Using the title ‘Pharmacogenomics Alert’ and the DNA symbol can help make participants aware and distinguish this PGx alert from other types of alerts.	“I like that it says the ‘Pharmacogenomics Alert’ and then the picture of the DNA.”—MD02.
		Participants like PGx-CDS contents to be formatted using bullet points, bolding, and underlining.	“I like the bullets, I like the bold, the underlining.”—MD05.
	Negative	One participant (MD03) thought the color was too harsh.	“I think the color is really harsh. It definitely caught my attention and I think it’s easy to read the text on it. I just wish it was a little bit brighter.”—MD03.
		Directions for asking questions were confusing. Participants did not know what to do with the messaging system.	“If I were reading both of those, I would just call the phone number.”—MD02.
		Reference pages had too much information to make a quick decision.	Participant was confused on how to navigate within the ‘More info’ link and eventually gave up, seeming not to find it very useful. “Using my surgery brain, I’m just looking for the high yield colorful pictures.”—MD02.
		Phenotype presentation was hard to recognize—poor formatting in the lab result session.	“I read things kind [of] quickly and the ‘Poor metabolizer’ doesn’t jump out at me.”—MD06.
		It was not distinctive to recognize whether one or more genomics results were presented and discussed within the PGx-CDS.	“I actually missed them in the first scenario.” Participant was clear that the CYP labs were labs but missed them on the first BPA because there was only one line.—MD05.
		Participants only focused on the information presented in the pronounced yellow section, everything else was skimmed through.	“I am always running late [busy]. I might even not read the non-highlighted area… when I see this, I would just switch the prescription.”—MD09.
	Suggestions	Lab results presented in CDS were hard to recognize. Participants suggest using icons/symbols such as a test tube/beaker to help with recognition.	“[Some color to that area… some kind of test tube… or picture, something that shows that it’s a lab.” Participant would like to see some color or picture (gave an example of a test tube) added to the CYP labs area—MD01.
		Phenotype information such as ‘poor metabolizer’ should be bolded (or red) to grab attention.	Participant felt that the ‘poor metabolizer’ should be bolded as well, “[like] the ‘increased risk of adverse events [is bolded]”—MD06.
		Participants preferred to see lab results sooner, at the top of CDS, or they should be presented in the yellow box.	“[A]s opposed to the other one [BPA1]… it was harder to see this… ultra-rapid… I would want that more up high [at the top, in the pronounced yellow section of the alert].” Participant preferred to see the metabolizer status at the top, in the pronounced yellow section of the alert—MD06.
3. Workflow	Positive	A hard stop in the workflow is necessary for prescribers to read the contents of the PGx-CDS. Prescribers emphasized that they wouldn’t be able to remember all patient data and keep track of all “flags” for each patient.	“I like the fact that it clearly grabs your attention as soon as you try to order the medication… and there is an actual stop in the workflow.”—MD03. “Honestly, we get so many of these flags that we don’t really keep track of what’s been flagged on each patient.”—MD04.
	Negative	N/A.	N/A.
	Suggestions	For PGx related orders, participants prefer to see the PGx alerts repeat, if relevant. However, one participant (MD06) would like the alert to fire once for each gene–drug pair. That participant is concerned for alert fatigue if the alert goes off every time for the same gene–drug pair.	“I think it would be helpful to see [the BPA] again… there’s a huge chance of having different people ordering medications.” The participant would like the BPA to appear every time medication connected with BPA is ordered because different prescribers may be ordering the medication.—MD01. “I’d be worried about getting fatigued… so I think anytime I add a new drug I think it is appropriate if it applies but dosing changes, I don’t think I would want them to pop up every time.”—MD06.
		Provide a link to the PGx results in the results review section in the EHR as some participants are not familiar with where they are located.	“I don’t necessarily know how to find [PGx results].” The participant would like a link to help explain the labs more.—MD03.
		One participant is interested in an option to document changes they made based on CDS in the EHR.	“I want [this] information to retain in EPIC^®^ . It gives me an opportunity to document my change… I like to document for myself from PGx result.” The participant asked where the information goes after they put in the medication order.—MD10.
4. Content	Positive	Alternative recommendations were useful when participants were not familiar with the different medication options.	“I really liked [BPA2] because it gave me multiple options.” Participant is referring to the recommendations section on BPA2, providing examples of medications they may select for each medication class option.—MD04.
		CDS problem statement directly told participants the risk for the gene–drug pair to know what to monitor.	“It actually tells you what the risk is, so that way you would know what to monitor the patient for if you were going to proceed in prescribing the medication.”—MD03.
		Providing phone numbers was helpful to get more information if needed.	“I like the fact that there is also a phone number there that I can call if I need to ask questions or get more information.”—MD03.
		Acknowledge Reason section is helpful and alerts the user that a change is needed.	“I like the fact that the ‘Acknowledge Reason’ included that I ‘Will adjust the dose’ as just another reminder that if I’m going to keep it that I need to do something different with it.”—MD03.
	Negative	Participants (MD02 and MD03) did not like ‘Disagree with recommendation’ and thought it should not be an option.	“I’d be curious to know who would select ‘Disagree with recommendation’.”—MD02. Interpreted ‘Disagree with recommendation’ as something to use for when there are medication shortages of other drugs. Participant didn’t think it would be a common choice.—MD03.
		Genotype information might not be useful.	“I don’t think the genotype is useful for me whatsoever, I’m not gonna know what the *3 *4 genotype actually means”—MD07.
		Providing genotype information is not helpful to making clinical decisions, and a shorthand description of ‘CYP2D6PHENO’ is confusing.	“I think it is confusing, the ‘CYP2D6PHENO’, I would change it to ‘PGx testing date’ and the result = ultra-metabolizer I think that would be more clear”—MD09.
		Information provided in the link is not useful to help describe the gene–drug pair.	“SSRIs metabolized by CYP enzymes, more information on physiology, possible interpretation of phenotypes to genotypes.” The participant expected this information in the ‘more information’ link—MD07.
		Adverse events listed due to the patient’s genotype are not specific enough, and giving examples would help explain the gene–drug pair.	When the participant was asked if listing the adverse events would have helped in his decision-making: ”I would want to know what to look out for as a reminder from medical school because that’s the last time I would have gone through the side effects.” Adverse effects were not listed in the second alert, unlike in the first alert.—MD06.
		Adverse events due to the patient’s genotype are too specific.	“Adverse reaction is really enough for me to change my prescription, whether it’s death or something else is not relevant.” The participant prefers a broader ‘adverse events may occur’ comment instead of a specific list of adverse events.—MD09.
	Suggestions	Keep genotype and phenotype lab results in CDS.	“I want to know the actual genetic phenotype and genotype.”—MD10.
		Acknowledge reasons should be more specific (for example, include an option to state ‘dose change’ if CDS recommends adjusting dose).	“If a lower dose would be considered safe, I would have that option [will adjust dose]… depending on the situation I guess, sometimes you absolutely cannot prescribe it.” The participant seems to like the ‘I will adjust dose’ option—MD09.
		Participant suggested adding a button to request a consult from the PGx team from CDS interface.	“One thing that would be great here, essentially this PMP monitoring, I know you can request a consult but this doesn’t tell you can request a consult if you’re not sure what the best option is.”—MD10.
5. Understandability	Positive	‘Problem statement’ and ‘Recommendations’ language in the alert is clear and easy to understand.	“What I get from that is… she does not metabolize it as fast or basically different from other people, so she [is] basically more sensitive.” The participant is referring to BPA wording.—MD02. “The [pronounced] yellow box [‘Problem statement’ and ‘Recommendations’ included here] gave me enough information for me to know what I should act upon, so I didn’t think I needed to click on that [more information] link.”—MD07. The participant states that information regarding the flag is clear and easy to understand.—MD04.
		Acknowledgment language was easy to understand.	“‘Will adjust dose’ makes sense because you can decrease it by 50%.” Participant thinks that options for ‘Acknowledge Reason’ makes sense, particularly with ‘Will adjust dose’ for BPA2 due to option to adjust dose in recommendation.—MD05.
	Negative	Difficulty interpreting and understanding the PGx labs (mainly by providers who have not seen PGx before).	“I didn’t realize necessarily that they were [CYP] labs. I’m not familiar with these phenotypes and genotypes.”—MD01. “She [the fictitious patient] is a *3/*4. I’m not totally sure what that means.”—MD02. “It’s very subtle… ultra-rapid metabolizer… I don’t know if that’s a severe or significant diagnosis vs. positive or negative”—MD02.
		‘P RX UF PMP MONITORING’ message is unclear. Participants do not understand who receives these messages and what the next steps are in the process. Participants are confused with the messaging system in EPIC^®^ (i.e., haiku, canto).	“If I were reading both of those [messages], I would just call the phone number.” The participant stated that the message “P RX UF PMP MONITORING” was confusing. The participant did not know what it meant or how to use it, and stated he would just call the phone number if he had a question.—MD02. “I have not used the EPIC’s^®^ messaging… it is not clear how to use it.” The participant stated they would just call the number if they had questions—MD09.
		Trouble understanding ‘Advisory sent via in basket message’.	The participant did not know what ‘This advisory has been sent via in basket’ meant—MD02. The participant stated that it does not say who it gets to or what happens when you message them—MD10.
		Participant not sure what “pheno” means, referring to the phenotype under lab results in the alert.	“What is PHENO”—MD09.
		Gene–drug pairs that have more than one genotype/phenotype are difficult to understand.	“CYP2D6? I thought we were talking about CYP2C19… so, the patient has both problems?” The participant was confused seeing CYP2D6 information when the alert is about CYP2C19. The participant stated that the alert ‘Problem statement’ is not clear regarding information with 2D6: “I don’t know the different between genotype and phenotype… I mean I know what they mean… how is the genetic testing different? I thought you just get a result.”—MD09.
	Suggestions	Make the metabolizer status association with the gene–drug pair clearer.	“I would want both of those to be… would want to say this person is a CYP2C19 poor metabolizer and CYP2D6 intermediate metabolizer… poor metabolizer status is associated with x, intermediate metabolizer status, right now CPIC doesn’t say anything about it… more likely to have adverse side effects down the line so that would also impact these choices”—MD10.
		Recommend removing genotype information (due to it being confusing and not understanding the value of having it in the alert).	I honestly again skimmed past it… the problem part in the very top was what I focused on… I don’t think the genotype is useful for me whatsoever, I’m not going to know what the *3 *4 genotype actually means.”—MD07. Phenotype makes sense to the participant, but genotype not as much. The participant stated that it is helpful to have it there for people who know more about the topic, such as about PM/IM/UM etc. and it needs to be in the pronounced yellow section at the top.—MD10.
6. Medical usefulness	Positive	BPAs were helpful at reminding the prescribers about PGx considerations such as potential adverse events.	The participant stated the following regarding the overall BPA: “the information is fantastic… I think really has the potential to cut down on adverse effects.”—MD01.
		Recommendations provided in the alert helped to make a clinical decision.	“It [the BPA] definitely made me think. It made me go back and change my order.”—MD01 “This message has me thinking as to what I should do [entire BPA]”—MD02. “We talk about pharmacogenomics and things a lot. We don’t take it into account all the time when prescribing, so I think personally this was a very useful message for me to come across.”—MD07.
		Alerts should fire off every time to help improve prescribing of PGx medications.	When participant was asked if they would like to see the alert every time the BPA can be applied: “Every time, because people forget.”—MD08.
	Negative	Not listing the adverse effects makes it difficult to determine the clinical importance.	“It [the BPA] tells you there’s just adverse effects, but it doesn’t really say what [those effects are]… I think it would be better if they can add on the actual adverse effects.”—MD08. “I would want to know what to look out for as a reminder from medical school because that’s the last time I would have gone through the side effects.” The participant stated this referencing those potential adverse effects that were not listed in BPA2, unlike BPA1.—MD06.
	Suggestions	Recommending alternatives for opioids would be more useful for decision making.	The participant states that an order set link would be helpful—MD05.
		Adding brand and generic would be more helpful for recognizing medications.	The participant thinks MDs would be more likely to choose an alternative they recognize (referencing the need for brand and generic to be listed)—MD05. “Like Dilaudid instead of Hydromorphone… people might not be as familiar with the non-brand name.” The participant recommends to consider putting the more common name used in practice for the medications or brand plus generic.—MD05.
7. Navigation	Positive	BPAs specific to PGx were not different compared to other BPAs that providers usually see, making the BPAs easy to navigate.	The participant stated: “that’s standard for how I currently use EPIC^®^ .” referring to not having issues with the remove and keep section—MD07.
	Negative	Order of how acknowledge reasons generate is confusing and hard to follow.	“I didn’t have too many thoughts on it because I decided to seek an alternative treatment… if I had decided to keep the medication, I probably would have looked more closely at it.” The participant ignored the ‘Acknowledge reason’ section because they chose to remove the order.—MD03.
		Leaving a comment after choosing your ‘Acknowledge’ reason is confusing and participants are unsure of what information is needed.	“So, I am not really sure what comment they want me to enter.”—MD03.
		Scrolling is required to see select alerts, which may lead to confusion on how to proceed.	“I just didn’t scroll down far enough to trigger the accept button to light up so that was the only thing I didn’t know why I would need to write something in addition to selecting the ‘Will adjust dose’.”—MD06.
	Suggestions	Acknowledge section needs to be more flexible and responses to the action taken by users.	“Maybe hide the ‘Acknowledge Reason’ unless you select ‘Keep’…because you don’t really need to acknowledge it if you’re removing and changing.”—MD04. “I intuitively felt like I need to acknowledge it with a button… The only button that I was looking for was like a ‘recommendation acknowledged- will remove order’.”—MD02.

## Data Availability

The data from this article cannot be shared publicly in order to maintain the privacy of individuals that participated in the study. The data will be shared upon reasonable request to the corresponding author.

## Supplementary Materials

The following are available online at https://www.mdpi.com/article/10.3390/jpm11111227/s1, Supplemental Material File S1: Case scenarios used during the usability session, Supplemental Material File S2: Usability script to guide the 60-min session.
