# Supplementary material for: Utilizing a Human–Computer Interaction Approach to Evaluate the Design of Current Pharmacogenomics Clinical Decision Support
Source: J Pers Med. 2021 Nov 18;11(11):1227. doi: 10.3390/jpm11111227 (PMC8618963; doi:10.3390/jpm11111227)
Supplement: Supplementary file 1 [file jpm-11-01227-s001.zip › jpm-1449054-supplementary.pdf]

Supplemental Files

Supplemental File S1 Case scenarios from usability session

Scenario 1

PMPNine PGx is a 70-year-old female with recent diagnosis of acute pain. After initial assessment, you decide to prescribe hydrocodone-acetaminophen (NORCO) to this patient.

Past medical history: no concern

Reviewing E-FORCSE indicated that this patient has not been prescribed hydrocodone-acetaminophen for the last 2 years.

Scenario 2

PMPfive PGx is a 53-year-old AA female who has recently been diagnosed with major depressive disorder and binge eating disorder.

Patient reports feeling lethargic, uninterested in her normal activities, and frequent episodes of crying throughout the week. She also endorses symptoms of binge eating disorder including anywhere from two to three binge episodes per week. She reports this then makes her feel more depressed.

She presents today to psychiatry for medication management and escitalopram is ordered.

Supplemental File S2 Usability script

Zoom setup

- Make sure the Session is recorded and saved locally
- Check functions that allow user to control your laptop through zoom
- Make sure you share the desktop, not just one software/app so the participant can change between different applications

Review Inform consent with participant,

Introduction

Today I will be reading from a standardized script to maintain consistency across participants. This is a research study to assess BPA that helps healthcare providers prescribe medications with pharmacogenetics information. Your participation in this ~60 minutes session will help us improve the design of the BPA.

As you complete the 4 tasks in our session today, please try to work on them as though they are part of your normal work tasks. However, if some tasks are slightly outside of your normal scope of practice, this is ok for our study today. Since this is a simulation study, you may feel as though you have less autonomy compared to your normal clinical work, and some things may feel more awkward or time-pressured. To complete the tasks, you will access a specific test patient chart in Epic EHR.

Please be honest in your feedback- you will not hurt anyone's feelings. We are evaluating the tool, not you or your clinical knowledge. Please 'think aloud' as you work on the tasks, by verbalizing your reactions, thought processes, emotions, and things you like or dislike about the tool. "Thinking aloud" may seem unnatural or uncomfortable since you are used to working silently. If you forget, I will gently remind you to verbalize what you are thinking.

Now I am going to show you an example of the Think Aloud Procedure. Please click the link in the upper right-hand corner of your screen. [Watch video].

What question do you have?"

Collect demographics if participant has not done it prior to the session

[Open a Redcap demographics survey. The survey should have these information below]

Participant #________________

Date of Session:_______________________

Demographics and other participant information

1. Gender
2. Degrees/certifications? (MD, PharmD, RN, etc)
3. What is your position and in what clinic?
4. How many years have you worked in a clinical capacity?
5. How long have you worked at the UF?
6. What year were you born?
7. What best describes your race/ethnicity?
8. How long have you used Epic EHR?
9. In an average month, how often do you encounter pharmacogenomics BPA?
10. Do you have any uncorrectable visual impairments or color blindness? Please describe.

[If yes: Please let me know if you have any trouble viewing or looking at anything on the computer today].

Instructions for usability tasks

I also understand that as a medical professional, your pager or cell phone may go off for an emergency. If this happens, please let me know how you would like to proceed (pause the session, stop the session, or reschedule).

In this session, we will give you the control of our screen through zoom, you will have access to all files located in the screen as well as the logged in EHR using our test provider.

Before we start the session, I would like to ask your permission to record this session.

[make sure you get verbal agreement from participant]

Please remember to read tasks aloud and think aloud as you go. During the session, you may ask questions and I will purposefully not respond so that I avoid biasing the session.

****

The cases provided are hypothetical cases with test patients. For the research session today, please use your Epic access to process your medication orders process. Since these are test patients, NO MEDICATION ORDERS will be sent to any pharmacy or patient.

I will log you in as a physician. The EHR might look slightly different than your daily EHR but you should be able to find all the functions needed for today's session. If you have problem finding a certain function, please let us know.

Task 1

Please read the patient case assigned to you. Follow the steps to order the recommended medication as if you were taking care of this patient in clinical practice at your workstation.

During the process, you will encounter a pharmacogenomics clinical decision support. Please review, take your actions, and use "think aloud" technique to provide your feedback.

Please state "I am done with case 1" when you are finished.

Task 2: Similar to tasks 1, please process with a second patient case assigned to you from the file/folder.

Task 3: Please complete the CSUQ survey provided. Please let me moderator know when you are finished.

Task 4: Please explain to the team what your concerns are (if anything) with the interface and the design of the pharmacogenomics BPAs you encountered and what steps you will take to remedy any issues.

[Ask these questions as relevant to the session]

1. Are there any missing information from the BPAs that you would like to see?
2. What suggestions do you have to help us improve these PGx BPAs?
3. Revisit scenario 2 and ask if participant can go through alternative options provided from the BPA.
    a. Ask participant if they have any comments related to alternative options
4. Ask participants about acknowledgement options? What concern or clarification they have with those options?

5. If we have a CDS that provide information regarding a chronic condition (for example CYP2C19 for long term PPI use), would you prefer it? Where should it be displayed?

This concludes this portion of the session. Please let the moderator know when you are finished. [end the record]

Do you have any other questions or comments for me at this time? [If no] Thank you so much for participating in our research study. [Give incentive gift]. If you have any questions or follow up questions after you leave, please feel free to reach out to our lead researcher Dr. Khoa Nguyen at Nguyen.khoa@cop.ufl.edu. His contact information is on the consent form.

Post usability session

Review and check all recorded file, redcap surveys. Video will take sometime to render, make sure the rendering processed is completed and copy the zoom folder to the shared drive

Check if the recorded file work as expected

Document the session in tracking excel sheet: participant ID, moderate name, time/date

Save consent form (electronic and paper) in appropriate locations

Supplemental File S2 Provides the document used to help guide the moderators when speaking with the healthcare providers through the usability session
